# Supplementary material for: Attenuation of Inflammation and Leptin Resistance by Pyrogallol-Phloroglucinol-6,6-Bieckol on in the Brain of Obese Animal Models
Source: Nutrients. 2019 Nov 14;11(11):2773. doi: 10.3390/nu11112773 (PMC6893810; doi:10.3390/nu11112773)
Supplement: Supplementary file 1 [file nutrients-11-02773-s001.pdf]

Supplementary material

Table S1. List of primer for Quantitative polymerase chain reaction (qRT-PCR)

Gene		Primers
CD11b	Forward	5'-TCAACACAACCAGAGTGGATTC-3′
	Reverse	5'- CCTAAAACCAAGCTTTGGACAC-3′
CD86	Forward	5'-AGCAACTAATGCTGAAAGCACA-3′
	Reverse	5'-CTCAGACCTGCCAAAGTTCTCT-3′
CD206	Forward	5'-TGTATTCTTTGCCTTTCCCAGT-3′
	Reverse	5'-GATAAAAGCCAGAAGCAGGAGA-3′
TNF-α	Forward	5'-TTCTGTCTACTGAACTTCGGGGTGATCGGTCC-3′
	Reverse	5'-GTATGAGATAGCAAATCGGCTGACGGTGTGGG-3′
IL-6	Forward	5'-CTGCAAGAGACTTCCATCCAGT-3′
	Reverse	5'-TCCTCTGTGAAGTCTCCTCTCC-3′
PPARγ	Forward	5'-TGGCAAAGCATTTGTATGACTC-3′
	Reverse	5'-ATTTGTCCGTTGTCTTTCCTGT-3′
ACC	Forward	5'-AGCTAACCCAACTCAGCAAGAC-3′
	Reverse	5'-GCAAATGGGAAGCAATAAGAAC-3′
FAS	Forward	5'-CTCCGAGAGTTTAAAGCTGAGG-3′
	Reverse	5'-GGGCCTCCTTGATATAATCCTT-3′
TLR4	Forward	5'-ATTCAGAGCCGTTGGTGTATCT-3′
	Reverse	5'-TCAAGGACAATGAAGATGATGC-3′
PERK	Forward	5'-CATCAGCACTTTAGATGGACGA-3′
	Reverse	5'-AGATGAAACCAAGGAACCAGAC-3′
eIF2a	Forward	5'-GAAGTGCCTAGTGAGGAGCCTA-3′
	Reverse	5'-CTCTTCATGCAGTTTGGAATTG-3′
IRE1	Forward	5'-ATCTGAAAAGGTTCCGCTCATA-3′
	Reverse	5'-TAGTGGTGCTTCTTGTTCCTCA-3′
Xbp1	Forward	5'-TTGAGGAAGCACCTCTAAGCTC-3′
	Reverse	5'-GGATGAAGTCATCTTCCAAAGG-3′
CEBP	Forward	5'-AGAAGTCGGTGGACAAGAACAG-3′
	Reverse	5'-GTTGCGTTGTTTGGCTTTATCT-3′
CD11c	Forward	5'-AGAACTGTTCACCACCCAAAGT-3′
	Reverse	5'-CAAGTTGTCCCCTTGTTTTCTC-3′
b-actin	Forward	5'-ACAAAGCTGTTCAGTGTCTCCA-3′
	Reverse	5'-CTCCGTTTCCAGAATACACACA-3′

Table S2. List of antibodies used in Immunoblotting

Antigen (host)	Company	Cat. No	Dilution rate
β-actin (rabbit)	Abcam	ab8227	1:5,000
NF-κB (rabbit)	Cell signaling	8242S	1:1,000
SOCS3 (mouse)	Santa cruz	sc-73045	1:1,000
STAT3 (mouse)	Cell signaling	#9139	1:1,000
pSTAT3(mouse)	Santa cruz	sc-8059	1:1,000
Ob-R (rabbit)	Abcam	ab5593	1:2,000

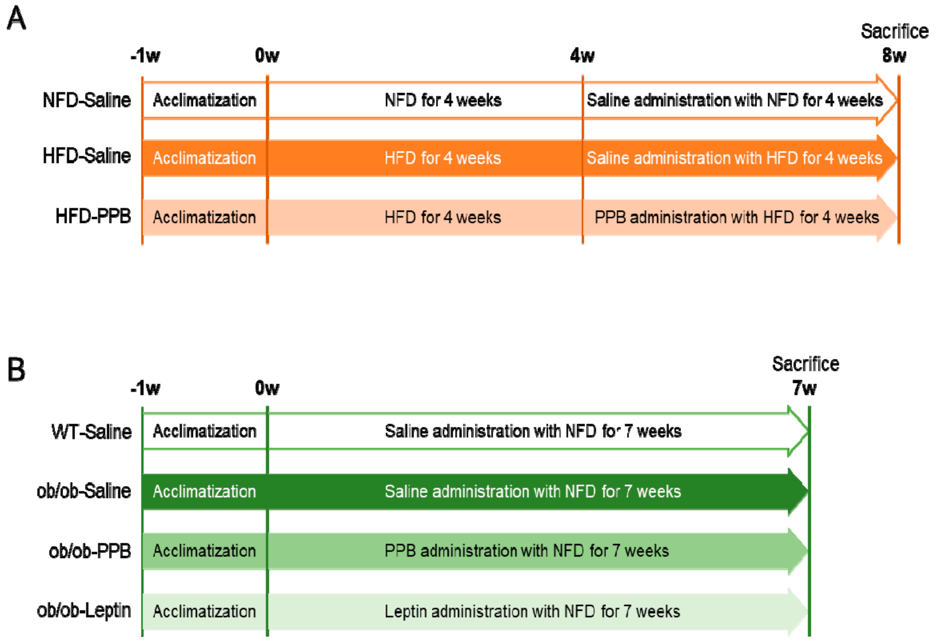

Figure S1. Experimental scheme image for animal modeling. Experimental scheme showed anima l modeling of diet induced obese mice (DIO) model and leptin deficiency mice (ob/ob) model. (A) To make DIO model, normal fat diet (NFD) or 45% high fat diet (HFD) fed for 4 weeks to C57BL/6 male mice, then 0.9% Saline (HFD-saline) or 2.5 mg/kg PPB (HFD-PPB) was daily administrated with the HFD next 4 weeks. All animals were sacrificed 8 weeks after the start of the experiment. (B) To make ob/ob model, wild type (C57BL/6) mice and ob/ob mice fed NFD for 7 weeks. During 7 weeks, 0.9% Saline (ob/ob-saline), 0.85 mg/kg/day Leptin (ob/ob-Leptin) or 2.5 mg/kg/day PPB (ob/ob-PPB) was daily administrated with the NFD. All animals we re sacrificed 7 weeks after the start of the experiment.

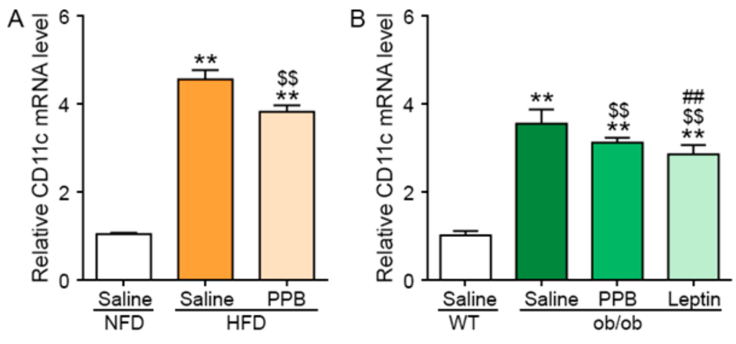

Figure S2. mRNA expression of CD11c as adipose tissue macrophages in visceral fat tissue. mRNA levels of CD11c as a adipose tissue macrophage marker in (A) visceral fat of DIO mice model and (B) ob/ob mice model were measured by qRT-PCR. All mRNA levels are expressed as relative levels normalized to β-actin of the NFD-Saline group or WT-saline group. Significance represented as **, $p < 0.01$ versus NFD-Saline or WT-saline; $$, $p < 0.01$ versus HFD-Saline or ob/ob-Saline; ##, $p < 0.01$ versus ob/ob-PPB. PPB, pyrogallol-phloroglucinol-6,6-bieckol
